# A Multi-Task Ensemble Strategy for Gene Selection and Cancer Classification

**DOI:** 10.3390/bioengineering12111245

**Published:** 2025-11-13

**Authors:** Suli Lin, Zhizhe Lin, Jin Zhang, Man-Fai Leung

**Affiliations:** 1 School of Cyberspace Security, Hainan University, Haikou 570228, China; 2 School of Computing and Information Science, Faculty of Science and Engineering, Anglia Ruskin University, Cambridge CB1 1PT, UK

**Keywords:** gene expression-based tumor classification, gene selection, multi-task ensemble strategy

## Abstract

Gene expression-based tumor classification aims to distinguish tumor types based on gene expression profiles. This task is difficult due to the high dimensionality of gene expression data and limited sample sizes. Most datasets contain tens of thousands of genes but only a small number of samples. As a result, selecting informative genes is necessary to improve classification performance and model interpretability. Many existing gene selection methods fail to produce stable and consistent results, especially when training data are limited. To address this, we propose a multi-task ensemble strategy that combines repeated sampling with joint feature selection and classification. The method generates multiple training subsets and applies multi-task logistic regression with $\ell_{2,1}$ group sparsity regularization to select a subset of genes that appears consistently across tasks. This promotes stability and reduces redundancy. The framework supports integration with standard classifiers such as logistic regression and support vector machines. It performs both gene selection and classification in a single process. We evaluate the method on simulated and real gene expression datasets. The results show that it outperforms several baseline methods in classification accuracy and the consistency of selected genes.

## 1. Introduction

Gene expression-based tumor classification is an important task in cancer research. It involves identifying tumor types using gene expression profiles. These datasets often contain tens of thousands of genes, which leads to very high-dimensional feature spaces. At the same time, the number of samples is usually small. This combination creates major challenges for analysis. To reduce complexity and improve performance, it is important to select a smaller subset of genes that carry the most useful information. Good gene selection can improve classification accuracy and also make the models easier to understand [1,2,3,4]. Finding patterns in gene expression data linked to specific cancer types can also help with diagnosis, prognosis, and designing targeted treatments.

Many gene selection methods have been proposed. They are usually grouped into three main types [5,6,7]: filter methods, wrapper methods, and embedded methods. Filter methods select genes before training the model. They do not depend on any specific classifier [8,9]. These methods use statistical scores to evaluate each gene, so they are fast and work well with high-dimensional data. Common examples are ReliefF and mRMR. ReliefF [10] assigns scores to genes based on how well they separate samples from different classes, using distance to nearby samples. mRMR (Minimum Redundancy Maximum Relevance) [11] selects genes that are both highly relevant to the class labels and not redundant with each other.

To combine the strengths of different measures, Zhang et al. [12] proposed a hybrid method that mixes ReliefF and mRMR. Omuya et al. [13] combined principal component analysis with information gain. Gong et al. [14] created a filter method that uses both Pearson correlation and mutual information. These hybrid approaches improve performance by selecting features that are both informative and diverse. In other areas, such as neurological disorder detection, hybrid methods have also shown strong results [15].

While filter methods are fast and scale well, they require users to set the number of selected genes in advance. This often needs expert knowledge. Also, since these methods run before model training, they do not adjust based on the classifier. This can reduce their performance, because the selected features may not match the model’s needs.

In contrast to filter methods, wrapper methods use a specific learning algorithm to evaluate different gene subsets. These methods run an iterative search to find the subset that gives the highest classification accuracy [16]. For example, Ghosh et al. [17] proposed a recursive memetic algorithm that refines gene subsets in steps to improve classification. GeFeS [18] is a genetic algorithm-based approach that uses multiple populations and adaptive weights to perform selection in high-dimensional settings. Other recent work includes a Markov blanket-based wrapper method by Hassan et al. [19], and a two-stage greedy search method for sentiment classification developed by Saugbacs and Arif [20].

Compared to filter methods, wrapper methods often reach higher classification accuracy because they directly test gene subsets during model training. However, they are slower and more computationally demanding. They are also more likely to overfit because the model is evaluated many times. To reduce these issues, some researchers have combined filter and wrapper methods. For instance, Sun et al. [21] combined ReliefF with ant colony optimization to improve tumor classification. Hu et al. [22] proposed a method that combines information gain, the Fisher Score, and evolutionary computation to perform efficient feature selection.

Embedded methods take a different approach. These methods include gene selection as part of the model training process. This makes them more efficient and often more generalizable. They usually rely on regularization to force sparsity in the learned weights, which helps interpret the selected genes [23,24,25,26,27]. For example, adaptive elastic net models select features by balancing relevance and redundancy during training [28]. Cawley et al. [29] used sparse logistic regression with Bayesian regularization to identify genes relevant to cancer, improving both accuracy and interpretability. Xing et al. [30] developed a hypergraph-based method that integrates gene selection into model learning using dictionary learning. Liu et al. [31] proposed a method that accounts for correlations between genes to improve selection and classification performance.

More recently, deep learning-based methods have been used for feature selection in gene expression analysis. These models can learn complex feature interactions during training and are well suited to high-dimensional data [32,33,34,35,36,37]. Deep learning models have also been used in related fields, such as medical image segmentation, where they are trained on noisy or AI-generated labels [38]. Other medical imaging studies show that learning texture-based patterns can improve diagnostic results. For example, Li et al. [39] used external image references to enhance MRI resolution and improve the visibility of fine structures. Similar methods have also been applied in plant disease detection using UAV images and an improved YOLO model [40].

Although many gene selection methods have been developed, most still struggle to produce gene sets that are both stable and biologically meaningful, especially when the data has high dimensionality and limited sample size. In gene expression–based cancer classification, this “high-dimension, low-sample” problem makes it hard to identify reliable biomarkers. Standard methods, including those based on evolutionary algorithms, are still widely used but often show inconsistent results and require careful parameter tuning. Deep learning and graph neural network (GNN) models have shown strong classification performance, but they usually need large datasets and still lack interpretability. Benchmarking practices for these models also remain unsettled.

To address these problems, we propose a multi-task ensemble strategy for gene selection in tumor classification. The method starts by generating multiple training subsets using random sampling. Each subset is then modeled using multi-task logistic regression with $\ell_{2,1}$ group sparsity regularization. This regularization, combined with ensemble sampling, helps identify genes that appear consistently across subsets, improving selection stability. Similar sampling-based strategies have been used in other domains to improve robustness and reduce bias during training [41]. The framework is general and can work with different classifiers; in this study, we use logistic regression. Because classification and gene selection are done together, the selected genes are more likely to be relevant across the dataset. This joint learning process improves both interpretability and classification performance compared to standard approaches.

The main contributions of this work can be summarized as follows:

- We propose a novel ensemble-based framework that systematically enhances gene selection stability by leveraging a sampling approach combined with multi-task learning.
- The use of $\ell_{2,1}$ regularization supports the selection of a compact yet informative gene set across multiple sampling subsets, reducing redundancy and improving model interpretability.
- Experimental results validate the effectiveness of our proposed ensemble approach, demonstrating that it achieves superior performance in terms of classification and gene selection consistency across datasets.

The remainder of this paper is organized as follows: Section 1 presents the proposed multi-task ensemble strategy for gene selection and cancer classification. Section 3 details the optimization procedure and provides the corresponding convergence analysis. Section 4 reports the experimental results and performance evaluation. Finally, Section 5 concludes the paper and outlines potential directions for future work.

## 2. Methodology

This section describes the proposed multi-task ensemble strategy for gene selection in tumor classification. The method combines data sampling with multi-task logistic regression and incorporates $\ell_{2,1}$ group sparsity regularization. The goal is to select genes that are both stable across samples and relevant for classification.

Let the gene expression data be $X\in R^{n\times d}$, where each row is a sample and each column is a gene. Let the label vector be $Y\in {\left\{-1,1\right\}}^{n}$. We first generate *m* training subsets by randomly sampling 70% of the data without replacement. This results in *m* separate tasks, with datasets $\left\{X^{1},X^{2},\ldots ,X^{m}\right\}$ and corresponding labels $\left\{Y^{1},Y^{2},\ldots ,Y^{m}\right\}$. Each subset is used to train a task-specific logistic regression model. The objective function is:

(1) $$\begin{array}{cc} \; & L(X,Y,W,c)\\ \; & =\sum_{k=1}^{m}\left(\frac{1}{n_{k}}\right)\sum_{i=1}^{n_{k}}log\left(1+exp\left(-Y_{i}^{k}\left(X_{i}^{k}W_{k}+c_{k}\right)\right)\right) \end{array}$$

Here, $W=[W_{1},W_{2},\ldots ,W_{m}]$ is the matrix of model weights for the *m* tasks, with each $W_{k}\in R^{d}$. The term $c_{k}$ is the bias for task *k*. Each $n_{k}$ is the number of samples in subset *k*, and $X_{i}^{k}$ and $Y_{i}^{k}$ are the *i*-th sample and its label in that subset.

**Figure 1:**
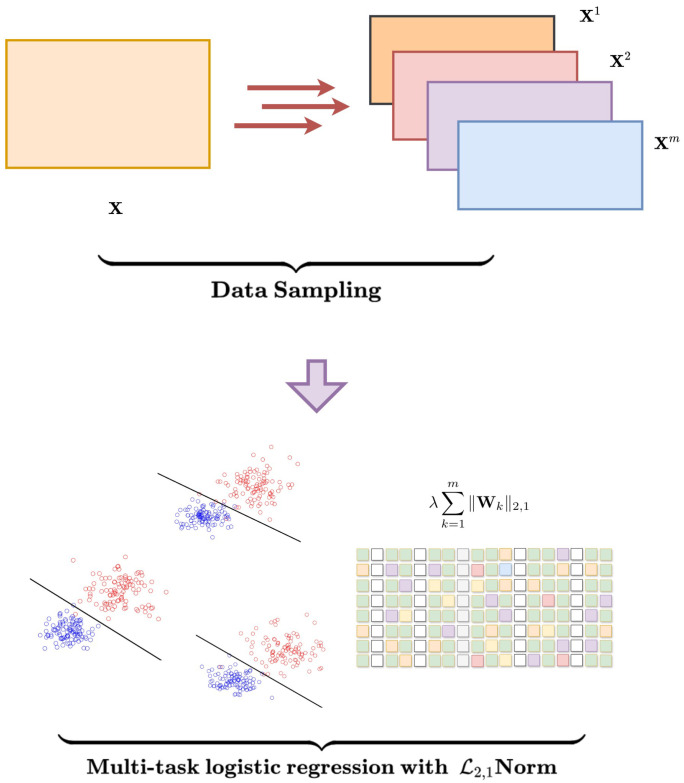
Diagram of the proposed multi-task ensemble framework. The pipeline consists of: (1) sampling multiple training subsets from the input gene expression data, (2) performing joint gene selection and classification using multi-task logistic regression with $\ell_{2,1}$ group sparsity, and (3) aggregating outputs to determine the final model. The colors are used only to distinguish different sampled subsets and task components, and they have no biological meaning.

In addition, the objective of this formulation is to select a set of relevant genes that are consistent across multiple tasks, while simultaneously maximizing overall classification accuracy. The goal is to identify a stable set of relevant genes that perform well in classifying tumor samples, ensuring that the selected genes are consistent and informative across all tasks. To achieve this, we utilize $\ell_{2,1}$ group sparsity regularization, which promotes shared gene selection across tasks. This regularization technique ensures that the genes chosen by the model are not only relevant to the classification task but also stable and consistent across different data subsets, enhancing the robustness and interpretability of the gene selection process. The Formulation (1) can be further defined as:

(2) $$\begin{array}{cc} \; & L(X,Y,W,c)\\ \; & =\sum_{k=1}^{m}\left(\frac{1}{n_{k}}\right)\sum_{i=1}^{n_{k}}log\left(1+exp\left(-Y_{i}^{k}\left(X_{i}^{k}W_{k}+c_{k}\right)\right)\right)\\ \; & +\lambda \sum_{k=1}^{m}{∥W_{k}∥}_{2,1} \end{array}$$

where $\sum_{k=1}^{m}{∥W_{k}∥}_{2,1}$ is $\ell_{2,1}$ group sparsity regularization and λ is a regularization parameter that controls the degree of sparsity. This $\ell_{2,1}$ norm encourages the selection of genes that are consistently relevant across tasks, thus promoting shared gene selection and ensuring that the model remains both efficient and interpretable. The overall processing of the proposed method is shown in Figure 1.

## 3. Optimization

In this section, we employ the proximal gradient descent method to optimize the proposed model. Specifically, we approximate the objective function in each iteration using a quadratic expansion around the current estimate. The approximation formulation for the weights W at iteration t+1 is given by:

(3) $$\begin{array}{c} \begin{array}{cc} \; & W(t+1)\\ \; & =min_{W,C}L\left(W(t)\right)+\langle W(t+1)-W(t),▽L(W(t))\rangle +\\ \; & \frac{\gamma }{2}{∥W(t+1)-W(t)∥}_{2}^{2}+\lambda \sum_{k=1}^{m}{∥W_{k}∥}_{2,1} \end{array} \end{array}$$

where γ is the step size, which can be determined by line search, and W(t+1) represents the updated estimate for W at iteration t+1. To further simplify, Equation (3) can be reformulated as:

(4) $$W\left(t+1\right)=min_{W}\frac{1}{2}{∥W-A∥}_{2}^{2}+\frac{\lambda }{\gamma }\sum_{k=1}^{m}{∥W_{k}∥}_{2,1}$$

where $A=\left(W(t)-(1/\gamma )▽L(W(t))\right)$. Overall, the following formulation allows for a closed-form solution for W(t+1) using the proximal operator associated with the $\ell_{2,1}$ norm, which promotes shared sparsity across tasks:

(5) $$W_{j}={\left(1-\frac{\lambda }{{∥A_{j}∥}_{2}\gamma }\right)}_{+}A_{j};\;\;\mathrm{for}\;\mathrm{each}\;\;j=1,2,\ldots ,d$$

The matrix W can be estimated using the above block-wise thresholding function.

Algorithm 1 shows the data sampling and optimization steps used to solve Equation (2). First, random sampling is used to create multiple subsets from the training data. These subsets define the tasks in the multi-task logistic regression model. Then, the weight matrix W is updated using proximal gradient descent. A line search adjusts the step size γ at each iteration. The algorithm repeats these steps until the objective function stops decreasing.
**Algorithm 1** Optimization1:**Input:** X,Y,λ, *m*2:**Initialize:** γ=1, η=0.7 (Samping rate)3:**Data Sampling:**4:**for** k=1:m **do**5:    Based on η=0.7, randomly generate subset index $\Gamma_{k}$6:    $X^{k}=X_{\Gamma_{k}}$7:    $Y^{k}=Y_{\Gamma_{k}}$8:**end for**9:**Proximal gradient descent:**10:**repeat**11:    Compute gradient ∇L(W(t)), set $A=(W\left(t\right)-\frac{1}{\gamma }\nabla L\left(W\left(t\right)\right))$12:    **for** j=1,2,…,d **do**13:         $W_{j}={\left(1-\frac{\lambda }{∥A_{j}{∥}_{2}\gamma }\right)}_{+}A_{j}$14:    **end for**15:    **if** $L\left(W^{\prime }\right)-L\left(W\left(t\right)\right)<\ldots$ **then**16:         **break** and output $W\left(t+1\right)=W^{\prime }$17:    **else**18:         γ=γ·α where α is user-defined19:    **end if**20:**until** convergence21:**Output:** W(t+1)

For λ-selection, we use 5-fold cross-validation over a logarithmic grid, select λ using mean validation log-loss (equivalently, maximizing validation accuracy), retrain on the full training subset with λ, and evaluate on the held-out test set. The same λ is shared across tasks to align sparsity patterns.

**Classification.** Our model jointly performs feature selection and logistic regression within a unified framework, enabling direct classification once the optimal parameters $\left(W^{★},c^{★}\right)$ are obtained. For a test sample $x_{i}\in R^{d}$, we define the task-specific logits as

$$z_{i}^{\left(k\right)}=x_{i}^{⊤}W_{k}^{★}+c_{k}^{★},\;k=1,\ldots ,m.$$

We then aggregate the predictions by averaging the logits across all *m* subsampled tasks:

$${\bar{z}}_{i}=\frac{1}{m}\sum_{k=1}^{m}z_{i}^{\left(k\right)}.$$

The aggregated logit is mapped through the logistic link function to produce the predicted probability:

$${\hat{p}}_{i}=\sigma \left({\bar{z}}_{i}\right)=\frac{1}{1+\exp(-{\bar{z}}_{i})}.$$

Finally, the predicted class label is assigned as

$${\hat{y}}_{i}=\left\{\begin{array}{cc} 1, & {\hat{p}}_{i}>\tau ,\\ 0, & {\hat{p}}_{i}\leq \tau , \end{array}\right.$$

where τ=0.5 by default.

**Vectorized form.** For a test data matrix $X\in R^{n\times d}$ and an all-ones vector $1\in R^{n}$, the prediction can be expressed compactly as

$$\hat{p}=\sigma \;\left(\frac{1}{m}\sum_{k=1}^{m}\left(XW_{k}^{★}+1c_{k}^{★}\right)\right),$$

where σ(·) is applied elementwise.

### Computational and Convergence Analysis

The proposed multi-task ensemble method uses random sampling, multi-task logistic regression, and $\ell_{2,1}$ group sparsity regularization. Optimization is done using proximal gradient descent. The total computational complexity is O(T·m·n·d), where *T* is the number of iterations, *m* is the number of sampled subsets, *n* is the number of samples, and *d* is the number of genes. The cost grows linearly with all key variables, so the method can scale to high-dimensional gene expression data. The $\ell_{2,1}$ regularization encourages shared sparsity across tasks, which improves the consistency of selected genes. The objective function is convex, so the algorithm converges to a global minimum. Related approaches using sparse regularization in multi-task settings have been explored for objective reduction in many-objective optimization problems [42,43]. Compared to filter methods, this approach is more computationally demanding but does not require setting the number of selected genes in advance and achieves better accuracy and stability.

Let $L\left(W\right)=f\left(W\right)+{\lambda ∥W∥}_{2,1}$ where f(W) is convex and has a Lipschitz continuous gradient with Lipschitz constant *L*. Let γ∈(0,1/L] be the step size. Then the following convergence results hold:

The sequence $\left\{W^{\left(t\right)}\right\}$ generated by the proximal gradient descent algorithm satisfies:
  - The objective value sequence $\left\{L\left(W^{\left(t\right)}\right)\right\}$ is non-increasing.
  - $L\left(W^{\left(t\right)}\right)\to L^{*}$, where $L^{*}$ is the global minimum.
  - Every limit point of $\left\{W^{\left(t\right)}\right\}$ is a minimizer of L(W).
Since f(W) is convex with Lipschitz continuous gradient, we have:
  (6) $$\begin{array}{c} \begin{array}{cc} \; & f\left(W^{\left(t+1\right)}\right)\leq f\left(W^{\left(t\right)}\right)+\langle \nabla f\left(W^{\left(t\right)}\right),W^{\left(t+1\right)}-W^{\left(t\right)}\rangle \\ \; & +\frac{L}{2}{∥W^{\left(t+1\right)}-W^{\left(t\right)}∥}_{F}^{2}. \end{array} \end{array}$$
Combining with the update rule using the proximal operator, we obtain that:
  (7) $$L\left(W^{\left(t+1\right)}\right)\leq L\left(W^{\left(t\right)}\right),$$

It shows monotonic decrease. By the descent property and the convexity of both *f* and ${∥W∥}_{2,1}$, standard results in proximal gradient methods ensure convergence to a global minimum. This convergence analysis guarantees that the proposed optimization procedure reliably finds a globally optimal solution under mild assumptions.

## 4. Experiment

This section presents the experimental results and analysis of the proposed method, evaluated on several simulated datasets and public microarray datasets characterized by high dimensionality and small sample sizes. First, the simulation and real gene expression datasets and the competing algorithms used in this analysis are briefly described. The experimental results are then presented and discussed from various perspectives.

### 4.1. Simulation Data

To simulate the high-dimensional nature of gene expression data, we generate high-dimensional datasets with small sample sizes. These datasets contain a large number of irrelevant features and a small set of relevant variables. Following the approach outlined in [31], the sample matrix is generated from a multivariate normal distribution. Specifically, we generate six different simulated datasets, each with varying sample and feature sizes. Each dataset includes 20 predefined relevant genes. Detailed information on the generated simulation data can be found in Table 1.

### 4.2. Real Gene Expression Data

This study evaluates the proposed method and competing approaches using four publicly available gene expression datasets:

- **Leukemia Dataset**: The preprocessed Leukemia dataset comprises 3571 genes and 72 samples, including 47 cases of acute lymphoblastic leukemia (ALL) and 25 cases of acute myeloid leukemia (AML).
- **Colon Dataset**: The Colon microarray dataset contains 2000 genes, with 22 samples from normal tissues and 40 samples from cancerous tissues.
- **Lung Dataset**: This dataset consists of 12,533 genes across 181 tissue samples, including 31 mesothelioma (MPM) samples and 150 adenocarcinoma (ADCA) samples.
- **DLBCL Dataset**: The used DLBCL dataset contains 6285 genes and comprises 58 samples of diffuse large B-cell lymphoma (DLBCL) and 19 samples of follicular lymphoma (FL).

Detailed information of the gene expression data can be found in Table 2.

### 4.3. Competing Methods

In this study, we compare the performance of the proposed method in a simulation setting with four baseline algorithms: the $L_{1}$-penalized model, the Elastic Net penalized model, mRMR with logistic regression, and ReliefF with logistic regression. Since mRMR and ReliefF are filter methods that select key genes before training the classification model, we set the number of selected genes to range from 10 to 50, with steps of 10 (i.e., [10, 20, 30, 40, 50]) for each dataset.

Both our method and the $L_{1}$-penalized model, as well as the Elastic Net penalized model, involve a parameter λ to control the sparsity of the model for gene selection and learning. Therefore, we use 5-fold cross-validation to automatically select the optimal parameter for the final model.

### 4.4. Experimental Setup

In our experimental studies, we randomly select 50% and 60% of the entire dataset as training data, with the remaining portion used as test data to evaluate the performance of each method. For each setting, the experiments are repeated 10 times, and the average results are reported.

### 4.5. Comparison Results

**Figure 2:**
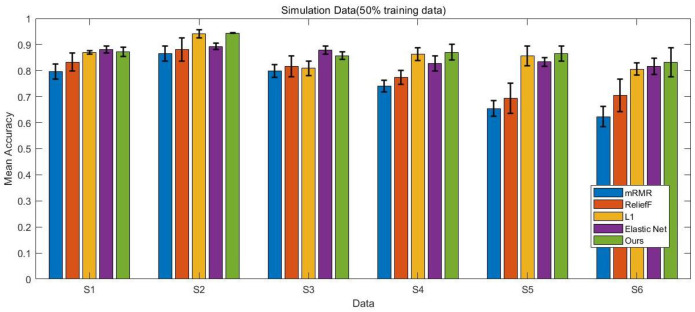
Prediction accuracy of five methods on six simulated datasets with 50% training data, comparing the proposed approach with baseline algorithms.

**Figure 3:**
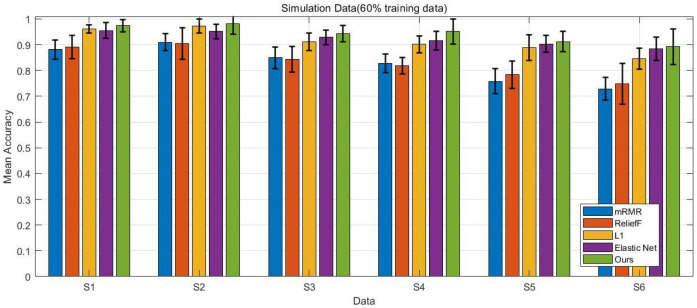
Prediction accuracy of five methods on six simulated datasets with 60% training data, comparing the proposed approach with baseline algorithms.

**Figure 4:**
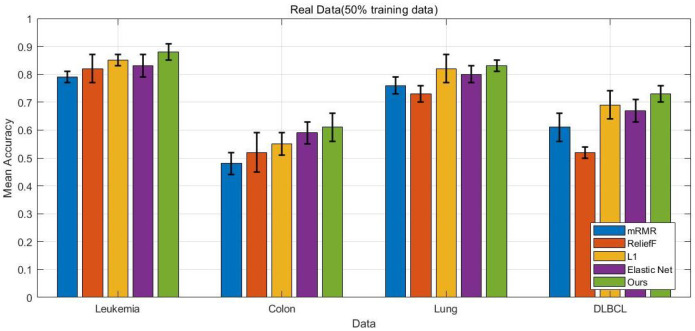
Prediction accuracy of five methods on 4 real gene expression datasets (Leukemia, Colon, Lung, DLBCL) with 50% training data, comparing the proposed approach with baseline algorithms.

**Figure 5:**
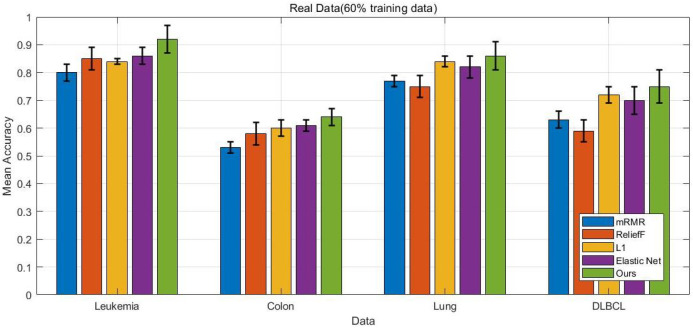
Prediction accuracy of five methods on 4 real gene expression datasets with 60% training data, comparing the proposed approach with baseline algorithms.

The experimental results presented in Figure 2, Figure 3, Figure 4 and Figure 5 provide a comprehensive comparison of the proposed multi-task ensemble strategy for gene selection against several baseline algorithms on both simulated and real gene expression datasets. The performance of each method was evaluated in terms of prediction accuracy using varying proportions of training data (50% and 60%). Specifically, Figure 2 and Figure 3 illustrate the prediction accuracy of the proposed approach and baseline algorithms on six simulated datasets with 50% and 60% training data, respectively. The results demonstrate that the proposed method consistently outperforms the baseline algorithms, including the $L_{1}$-penalized model, the Elastic Net penalized model, mRMR combined with logistic regression, and ReliefF combined with logistic regression. Similarly, for the real datasets, the proposed multi-task ensemble strategy exhibits superior performance, with particularly significant improvements in classification accuracy observed in the Leukemia and Colon datasets. Overall, models that integrate gene selection and classification learning, such as the $L_{1}$-penalized model, the Elastic Net penalized model, and the proposed multi-task ensemble strategy, generally outperform filter-based approaches like mRMR and ReliefF.

The results further highlight that the integration of multi-task learning and $\ell_{2,1}$ group sparsity regularization in the proposed strategy not only improves the interpretability of the selected gene subsets but also enhances the overall robustness of the classification models.

### 4.6. The Influence of Multi-Task Ensemble Learning Strategy

**Figure 6:**
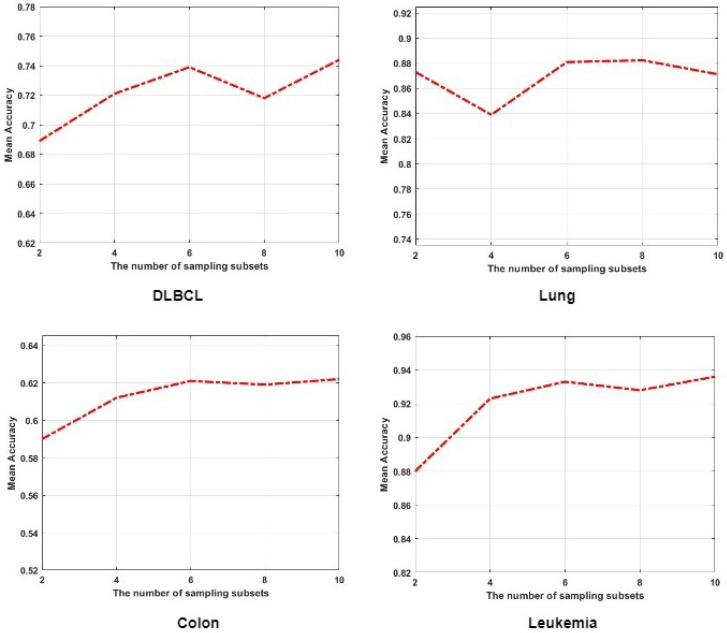
Effect of varying the number of sampling subsets (k=2,4,6,8,10) on classification accuracy. Results are averaged over multiple runs on four datasets.

The multi-task ensemble learning strategy is a key component of the proposed method. To further evaluate its effectiveness, we conducted experiments on four datasets. Specifically, we examined the performance of our method under varying numbers of sampling subsets *k* within the range [2,4,6,8,10]. The average accuracy (ACC) results are presented in Figure 6. From these results, it can be observed that our method achieves the best performance within the range [6,8,10], demonstrating that a sufficient number of sampling subsets enhances the model’s robustness. This further verifies the effectiveness of the proposed multi-task ensemble learning strategy, as it leverages complementary information from multiple subsampled tasks to reduce estimator variance and mitigate overfitting. By aggregating predictions across diverse tasks, the model captures consistent patterns shared among different data partitions, leading to more reliable and generalizable representations. Consequently, the ensemble structure not only stabilizes the learning process but also improves predictive accuracy across heterogeneous gene expression datasets.

### 4.7. The Effectiveness of Gene Selection

The primary objective of the proposed method is to identify relevant gene subsets. To evaluate its effectiveness, we report the mean number of selected genes and the mean number of relevant genes identified on the simulated dataset, comparing our method with competing approaches, as summarized in Table 3.

The ground truth number of relevant genes is 20. For mRMR and ReliefF, which are filtering-based methods, the number of selected genes must be predefined; in this evaluation, it is set to 100. The results show that, compared with competing methods, our approach effectively identifies most of the relevant genes (close to 20) across all settings while selecting a smaller and more meaningful subset of features. In contrast, mRMR and ReliefF rely solely on pairwise relevance measures between features and labels, which may overlook complex inter-feature dependencies or redundant information. These methods also require a user-specified number of selected genes, making their performance sensitive to this hyperparameter. In comparison, our method integrates feature selection and model learning within a unified optimization framework, enabling the adaptive determination of informative genes without prior knowledge of their number. This joint learning strategy not only reduces manual parameter tuning but also improves interpretability and biological relevance, as the selected genes are directly linked to the model’s predictive objective. Consequently, our method provides a more data-driven and principled approach than traditional filtering techniques such as mRMR and ReliefF.

### 4.8. Biological Analysis of the Selected Genes

To validate the biological interpretability of our proposed method, we conducted a detailed analysis of the top-ranked genes identified in the experiments. Table 4 summarizes the top five genes selected for the Colon and Leukemia datasets. Notably, all genes identified by our approach have also been reported in previous studies using established gene selection techniques, thereby confirming the biological relevance and robustness of our findings.

For the Colon dataset, for instance, the gene H06524 (Gelsolin precursor, plasma) was also reported by [44], and T94579 (Human chitotriosidase precursor) was identified by [45]. Similarly, for the Leukemia dataset, M23197 (CD33 antigen) was also selected by [46], further demonstrating the consistency of our results with well-recognized biological evidence.

## 5. Discussion

The proposed gene-selection strategy is both effective and stable. Our group-sparse multi-task formulation induces shared sparsity across tasks, yielding compact and interpretable panels. In simulation, it correctly recovers most of the 20 ground-truth genes while keeping the selected set comparatively small, and, unlike filter methods, it does not require pre-specifying the subset size.

Despite these strengths, two practical constraints remain—common to many gene-selection frameworks. First, the high-dimensional, low-sample regime can limit generalization even with ensembling. Second, our current evaluation uses only transcriptomic measurements. To address these, in the future, we plans to integrate multi-omic signals (e.g., RNA-seq, copy-number variation, DNA methylation, proteomics) via early/late fusion within the same multi-task objective to better exploit shared structure.

## 6. Conclusions

This study presents a novel multi-task ensemble strategy for gene selection in gene expression-based tumor classification, aiming to address the challenges posed by high-dimensional data and the demand for both effective and interpretable models. The proposed method integrates gene selection and classification within a unified multi-task logistic regression framework, incorporating $\ell_{2,1}$ group sparsity regularization to promote consistent and robust gene selection across multiple data subsets. Extensive experiments on both simulated and publicly available gene expression datasets demonstrate that the proposed approach consistently outperforms existing baseline methods in terms of classification accuracy and gene selection stability. By leveraging multi-task learning and structured sparsity, the model enhances both robustness and biological interpretability, offering a promising direction for gene expression analysis and cancer diagnosis.

## Figures and Tables

**Table 1:** Simulation dataset specifications. Each dataset consists of a specified number of samples and gene expression features.

Dataset	Sample Size	Feature Dimension
S1	100	500
S2	200	500
S3	100	1000
S4	200	1000
S5	100	2000
S6	200	2000

**Table 2:** Summary of real gene expression datasets used for evaluation.

Dataset	Sample Size	Feature Dimension
Leukemia	72	7129
Colon	62	2000
Lung	181	12,533
DLBCL	77	6258

**Table 3:** Average number of selected genes and, after the slash, the number of those that match the 20 relevant genes embedded in the simulated data. Format: selected/relevant.

Dataset	$L_{1}$	Elastic Net	mRMR	ReliefF	Ours
S1	12.3/21.6	16.6/28.9	14.2/50	11.8/50	18.1/27.2
S2	17.6/22.9	18.3/36.1	13.1/50	13.9/50	19.1/30.2
S3	11.4/27.6	15.3/32.4	14.9/50	12.4/50	17.8/26.4
S4	15.9/28.2	16.8/34.6	14.4/50	14.1/50	18.6/31.2
S5	12.1/35.1	14.8/52.7	12.1/50	10.9/50	17.8/30.4
S6	14.6/34.4	14.9/59.9	13.7/50	11.8/50	18.3/28.9

**Table 4:** Top five genes selected by the proposed method on the Colon and Leukemia datasets. All listed genes have been previously reported in the literature and are known to be biologically relevant to the corresponding cancer types.

Dataset	Gene ID	Description
Colon	H06524	Gelsolin precursor, plasma.
	T92451	Tropomyosin, fibroblast and epithelial muscle-type.
	H20709	Myosin light chain alkali, smooth-muscle isoform.
	T94579	Human chitotriosidase precursor.
	R88740	ATP synthase coupling factor 6, mitochondrial precursor (Human).
Leukemia	X62654	ME491 gene extracted from *Homo sapiens* antigen.
	M23197	CD33 antigen.
	M63138	Cathepsin D, lysosomal aspartyl protease.
	Y00787	Glutathione peroxidase 1.
	U05259	MB-1 gene.

## Data Availability

No new data were created or analyzed in this study.
